# Optimized Alkali-Activated Slag-Based Concrete Reinforced with Recycled Tire Steel Fiber

**DOI:** 10.3390/ma15196623

**Published:** 2022-09-23

**Authors:** Milad Eskandarinia, Mina Esmailzade, Ata Hojatkashani, Aida Rahmani, Soheil Jahandari

**Affiliations:** 1 Department of Civil Engineering, South Tehran Branch, Islamic Azad University, Tehran 15847-43311, Iran; 2 Department of Civil Engineering, Shahid Rajaee Teacher Training University, Tehran 16788-15811, Iran; 3 Centre for Infrastructure Engineering, Western Sydney University, Penrith, NSW 2751, Australia

**Keywords:** alkali-activated slag-based concrete, fiber-reinforced concrete, image processing technique, recycled tire steel fiber, Taguchi-Grey relational analysis method

## Abstract

This study employed Taguchi-Grey relational analysis to optimize the influences of binder content, the molarity of sodium hydroxide (SH) solution, alkaline solution to binder content (Al/Bi) ratio, water to alkali-activated solids (W/S) ratio, and sodium silicate to sodium hydroxide solution (SS/SH) ratio on the workability, setting time, and compressive strength of alkali-activated slag-based concrete (AASC). Then, the recycled tire steel fibers (RTSF) were introduced into the optimized mixture in different dosages, and the physical and mechanical properties of fiber-reinforced AASC (FR-AASC) were evaluated. RTSF inclusion negatively affected the workability and increased the density while slightly reducing the water absorption. Additionally, the compressive strength and flexural behavior of FR-AASC improved by increasing the RTSF content. The analysis of images taken from flexural specimens through the Digital Image Correlation technique (DIC) revealed that higher RTSF dosage caused a curved macro crack with several branches alongside, leading to a better post-cracking performance in terms of strength and toughness.

## 1. Introduction

The Portland cement industry is responsible for substantial carbon emissions [1]. The alkali-activated concrete (AAC), employing by-products of other industries as its cementitious material, is a green alternative to ordinary Portland cement concrete (OPC) and can reduce the carbon footprint by generating much less CO_2_ compared to OPC [2]. Additionally, some challenges related to the limited available landfills for dumping industrial waste materials such as slag can be ironed out by expanding AAC in the construction industry. The alkali-activated material, which has been occasionally termed as a geopolymer, comprises two main components, the source material based on aluminosilicate and an alkaline activator [3]. The aluminosilicate source, which can be selected from by-product materials like slag and fly ash [4], must be rich in alumina and silica [5]. Slag and fly ash have been widely utilized as raw materials in the preparation of AAC due to the availability, low cost, and high content of Si and Al [6]. A combination of sodium hydroxide (SH) and sodium silicate (SS) or potassium hydroxide and potassium silicate is the most common alkaline solution utilized in AAC [7].

Numerous studies have been conducted on salient factors which influence the alkali-activated mortar and concrete properties. Hanjitsuwan et al. [8] observed that when the SH concentration increased, the compressive strength and setting time of high calcium fly ash geopolymer paste were enhanced. Aliabdo et al. [9] stated that increasing the fly ash amount resulted in an improvement in the workability and compressive strength of fly ash-based geopolymer concrete. Aliabdo et al. [4] also reported that an increase in additional water, the sodium hydroxide solution to sodium silicate solution ratio, and the alkaline solution to fly ash content ratio, as well as the decrease in the molarity of SH, caused an enhancement in the workability of fly ash-based geopolymer concrete. Fang et al. [10] saw that by increasing the molarity of the SH and slag content, the workability and setting time of alkali-activated fly ash-slag concrete were reduced, while the compressive strength was increased. Hadi et al. [11] found that as the alkaline solution to binder (Al/Bi) ratio and additional water to binder ratio rose, the setting time and workability of ground granulated blast furnace slag-fly ash-based geopolymer concrete were improved, although the compressive strength was reduced. Nguyen et al. [12] declared that the replacement of low-calcium fly ash by GGBFS significantly enhanced the compressive strength, even though it slightly decreased the slump value.

Based on the above review, it can be easily understood that each parameter has different effects on the essential properties of AAC and constrains its actual utilization in the construction industry. So, the optimal mixture investigation to obtain the desired qualities of AAC is an essential issue. However, establishing an optimal mixture of AAC by traditional methods like full factorial design has two main problems of high cost and being time-consuming. Therefore, the Taguchi method, proposed by Genichi Taguchi during the 1950s, can be employed as a systematic optimization approach to reduce the overall testing time and experimental costs by decreasing the number of tests [13,14]. Olivia and Nikraz [15] conducted a study with the help of the Taguchi method to achieve an optimal fly ash geopolymer mixture in terms of compressive strength by considering the effects of the alkaline solution to fly ash ratio, the amount of aggregate, curing, and sodium silicate to sodium hydroxide (SS/SH) ratio. Hadi et al. [3] adopted the Taguchi method to determine the optimum level and percentage of the participation of examined factors (binder content, SH concentration, SS/SH ratio, and Al/Bi ratio) on the compressive strength of geopolymer concrete with ground granulated blast furnace slag. Jithendra and Elavenil [2] selected the Taguchi method to evaluate the influence of the percentage of coarse aggregate, the molarity of SS, the percentage of superplasticizer, and the solution-to-binder ratio on the compressive strength and slump flow of aluminosilicate-based flowable geopolymer and find optimum mix proportions.

It is also worth noting that control parameters can have different effects on different performances of AAC simultaneously. For example, by increasing the extra water, the workability improved while the compressive strength decreased [12], or increasing the concentration of SH had a positive influence on compressive strength and setting time. In contrast, it caused a reduction in workability [10]. Therefore, the optimal combination of controlled parameters determined for one characteristic may not be feasible for another characteristic. So, multi-response optimization seems to be crucial for designing the AAC mixture. However, the Taguchi is an unsuitable method for simultaneously optimizing multiple performance characteristics [16]. Thus, the Grey relational analysis (GRA), developed by Deng [17], is identified as an appropriate technique for resolving multi-objective optimization problems. It can be linked with the Taguchi method to obtain the optimum combination of examined parameters concerning multiple responses simultaneously [18,19]. To the best knowledge of the authors, only Prusty and Pradhan [20] employed Taguchi-Grey relational analysis to determine the optimum mix proportion of fly ash-slag-based geopolymer concrete by considering the slag replacement, the molarity of SH, the binder content, the water to geopolymer solids ratio, and the SS/SH ratio as parameters and compressive strength, workability, and setting time as examined responses.

In spite of owning excellent properties in terms of sustainability and compressive strength, AAC suffers from brittleness and sudden failure [21]. To put it in another way, the deficiency of low flexural and tensile strength is one of the weakest points in these materials [22]. However, this weakness can be addressed by adding fibers into the matrix. Extensive research has been performed to evaluate the influence of fibers on the properties of AAC [1,21,22,23,24]. A total of 60 million tons of different types of fiber, the production of which needs large amounts of raw materials and energy, are utilized annually to reinforce concrete [25]. Zhang et al. [26] investigated the impact of polyethylene fibers and the water-binder ratio on the fracture properties of high-toughness geopolymer by considering the fiber factor. They found out that polyethylene has a good toughening effect on geopolymer. Chao et al. [27] developed multiscale hybrid fiber reinforced cementitious composites with steel fibers, polyvinyl alcohol (PVA) fibers, and calcium carbonate whiskers with improved mechanical properties under static load. They demonstrated that the multiscale hybrid fibers act as a bridging ligament and resist against crack propagation under dynamic load, resulting in a longer time period with improved displacement, strain, and acceleration, and also proposed optimized mixtures for short- and long-length hybrid fibers. Li et al. [28] performed flexural strength tests on polypropylene fiber-reinforced geopolymer mortar. They collected the surface cracking images using a digital camera and extracted cracking information by deep learning. They found out that the crack length, width, area, and fractal dimension of the specimen increased when increasing the fiber volume fraction. Meanwhile, in recent decades, the world has experienced a surge in the number of end-of-life tires. It is estimated that the weight of scrap tires will reach 1.2 billion tons by the end of the 2030s [29]. Owing to the adverse environmental problems associated with the disposal of these waste tires (occupation of large areas, soil pollution, and releasing toxic fumes), the management of scrap tires has become a critical issue in recent decades. Reutilizing the internal steel reinforcement of tires, known as recycled tire steel fiber (RTSF), in concrete is a promising way to iron out the challenges mentioned above.

Several studies have been undertaken to assess the impact of employing RTSF as an alternative to industrial steel fiber (ISF) to reinforce mostly conventional cementitious concrete [30,31,32,33,34,35]. However, a thorough survey of the relevant scientific literature resulted in only three articles associated with the investigation of the effect of RTSF on the mechanical properties of AAC. Mucsi et al. [36] found that rubber-contaminated RTSF addition to the fly ash-based geopolymer concrete reduced the compressive strength, while pure steel fiber significantly increased it. They also reported that either pure or contaminated RTSF improved flexural strength. Zhong et al. [37] observed that the workability of the alkali-activated fly ash-slag mortar decreased by up to 32.8% when the RTSF content in the mixture increased. However, adding fiber significantly improved the flexural strength and compensated for the loss of compressive strength caused by crumb rubber inclusion. Wang et al. [38] reported a reduction in the workability value and a significant enhancement in the compressive strength value as a result of RTSF addition to the fly ash-slag-based strain-hardened geopolymer composites. The flexural strength experienced a negligible reduction at 7 days, although it increased at 28 days, compared to the plain mixture. Therefore, there still exists a large knowledge gap on the performance of RTSF in AAC.

Firstly, this study aims to produce an AASC with an optimum mix proportion by considering the effects of the most influencing parameters, including binder content (350, 375, 400, 425 kg/m^3^), the molarity of the sodium hydroxide solution (10, 12, 14, and 16 M), the alkaline solution to binder content ratio (0.40, 0.45, 0.50, 0.55), the water to alkali-activated solids ratio (0.35, 0.375, 0.40, 0.425), and the sodium silicate to sodium hydroxide solution ratio (1.75, 2.00, 2.25, 2.50) on workability, initial and final setting times, and compressive strength (after 7 and 28 days of curing). Secondly, RTSF with three different volume fractions (0.2%, 0.4%, and 0.6%) was added into the obtained optimal AASC mixture. Then, the physical and mechanical properties of the resulting fiber-reinforced alkali-activated slag-based concrete (FR-AASC), namely, workability, density, water absorption, and compressive strength were systematically investigated. The emphasis was placed on the flexural behavior of FR-AASC in terms of first-crack and residual strength, toughness, toughness indices, and residual strength factors. Crack propagation in bending specimens was also evaluated by the Digital Image Correlation (DIC) technique.

## 2. Research Significance

In light of the literature, the optimization of the mix proportions of alkali-activated slag-based concrete (AASC) with regard to multiple performances to develop AAC as an eco-friendly material is essential. Therefore, this study aims to make a significant contribution to the development of AASC by resolving the issues associated with rapid setting time and poor workability, which limit its field application in the construction industry. The novelty of this work is employing the Taguchi-Grey relational analysis method as a multi-objective optimization approach, which significantly reduces the number of experimental tests. Moreover, most of the published papers about recycled tire steel fibers (RTSF) are associated with the influence of this type of fiber on conventional cementitious concrete. On the other side, very few studies have focused on investigating the properties of AAC reinforced with RTSF. Thus, the second objective of the current research is to provide a comprehensive understanding of the effects of RTSF as a novel generation of reinforcement on the properties of AASC, which is considered as an economically and environmentally viable alternative for cement-based concrete.

## 3. Materials and Methods

### 3.1. Materials

#### 3.1.1. Slag

**Figure 1:**
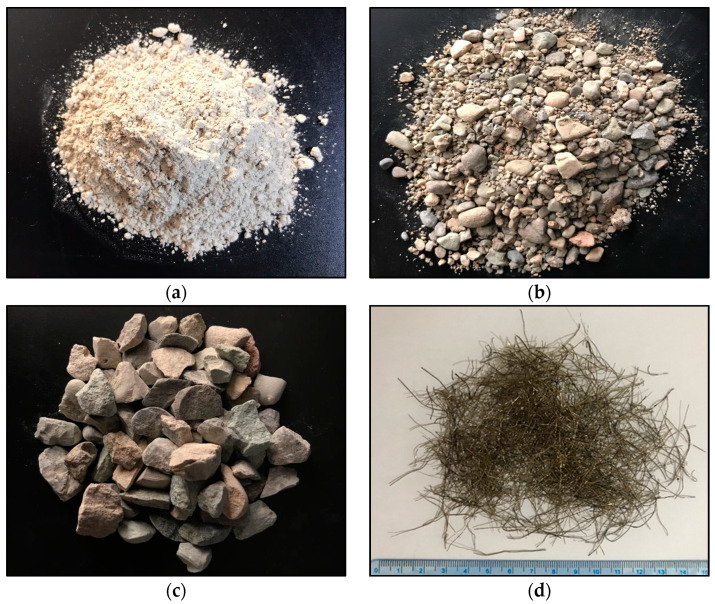
Raw materials (**a**) slag (**b**) fine aggregates (**c**) coarse aggregates (**d**) RTSF.

Slag (Figure 1a), supplied by Sepahan Cement Co. (Esfahan, Iran), was used as an aluminosilicate source material in this work. The specific gravity of slag was 3.002. The chemical compositions by X-ray fluorescence (XRF) spectrometry are illustrated in Table 1.

In order to calculate the hydraulic activity of slag, the basicity coefficient (K_b_ = CaO + MgO/SiO_2_ + Al_2_O_3_) was measured. With K_b_ = 1, the employed slag was classified as a neutral one, which is suitable as a starting material for an alkali-activated slag binder [39]. The hydration modulus (HM = CaO + MgO + Al_2_O_3_/SiO_2_), which is suggested to be greater than 1.4 to ensure good hydration properties [40], was calculated as 1.61. Additionally, the presented slag can be accepted as the binder material because the CaO/SiO_2_ and Al_2_O_3_/SiO_2_ ratios of chosen slag (1.05 and 0.3, respectively) were in the defined intervals of 0.5 to 2 and 0.1 to 0.6, respectively [5].

#### 3.1.2. Activators

The combination of Sodium silicate (Na_2_SiO_3_) solution and sodium hydroxide (NaOH) solution was adopted as the alkaline solution. Water quality has a significant effect on the mechanical properties of concrete and cementitious materials. The sodium silicate solution, containing 28.3% SiO_2_, 11.7% Na_2_O, and 60% H_2_O by mass, was selected from a local commercial producer. The SiO_2_/Na_2_O ratio, termed silica modulus, was calculated as 2.42. Commercially available sodium hydroxide flakes (98% purity) were dissolved in tap water to prepare a sodium hydroxide solution with desired concentrations.

#### 3.1.3. Aggregates

River sand was chosen as fine aggregate (Figure 1b) with a specific gravity of 2.45, a fineness modulus of 3.24, and 1.28% water absorption. The utilized coarse aggregate (Figure 1c) was crushed granite of 12.5 mm (maximum size) with a specific gravity of 2.57 and 1.91% water absorption. Fine and coarse aggregates, obtained from a quarry in Shahryar, Iran, were employed in saturated surface dry (SSD) conditions according to ASTM C128-15 [41] and ASTM C127-15 [42], respectively. The percentage of coarse aggregate was selected at 50%, which was determined by [2] as the optimal level with respect to compressive strength and workability and of aluminosilicate-based flowable geopolymer concrete.

#### 3.1.4. Superplasticizer

The commercially known P10N superplasticizer (SP) based on polycarboxylate ether with a specific gravity of 1.1 was utilized to enhance the workability of alkali-activated slag-based concrete (AASC). The dosage of 1% SP, published by [2] as an optimum percentage of SP for aluminosilicate-based flowable geopolymer concrete, was considered for the present research.

#### 3.1.5. Fiber

The recycled tire steel fibers (RTSF), shown in Figure 1d, supplied by a local recycling factory, were employed in three volume fractions (0.2%, 0.4%, and 0.6%) to reinforce the optimal AASC mix determined in Section 4.1. Based on the pilot tests, the challenge of the further addition of RTSF was an increased susceptibility to balling due to the irregular geometry of fibers and the high viscosity of the mixture. The obtained RTSF significantly varied in diameter and length due to the mechanical recycling process, i.e., the shredding process. Therefore, a statistical analysis was performed on a 15 g sample, revealing that the RTSF utilized in this paper had an average length and diameter of 16.20 and 0.23 mm, respectively. The average elastic modulus and tensile strength of RTSF were not measured, but according to [37], they are about 200 GPa and 2570 MPa, respectively.

### 3.2. Mixing, Casting, and Curing

The mixing process consisted of the following stages. The SH solution was prepared in the required concentrations (10, 12, 14, and 16 M) a day before mixing to allow it to reach the ambient temperature and to remove the negative effect of its excessive heat on the setting time [5,43,44]. After 24 h, the alkaline solution was first prepared by mixing SS and SH solutions around 30 min before the main mixing, which is suggested by [9] to enhance the properties of the geopolymer mixture. After that, coarse and fine aggregates were poured into the pan mixer and mixed for two minutes, then slag was added and mixed for another 2 min to have the homogeneity of the dry materials. Simultaneously, superplasticizer and extra water, which was tap water according to the authors’ previous research studies [45,46,47,48,49], were poured into the prepared alkaline liquid and thoroughly mixed with the help of a stirrer for 2 min to obtain a homogenous liquid component. Afterward, the liquid component was gradually added to dry materials, and mixing continued for approximately 4 min to achieve the uniform nature of concrete. It is also worth noting that, in order to prepare fiber-reinforced alkali-activated slag-based concrete (FR-AASC) in Section 4.2, after achieving a well-combined AASC, RTSFs were slowly added into the mixture to prevent the balling phenomenon.

The fresh concrete was poured into relevant molds in two layers, and each layer was compacted on the vibration table for 15 s. The samples were left in molds at the laboratory temperature of 20–23 °C for 24 h and covered with wet hessian fabric to avoid excessive water evaporation. The specimens were then de-molded and cured in plastic bags, as suggested by [50,51].

### 3.3. Testing Methods

In order to achieve the optimal mix design for AASC and investigate the physical and mechanical properties of FR-AASC, tests were conducted in two phases, described in the following sections.

#### 3.3.1. Optimization of AASC

The workability of fresh AASC was investigated by the slump cone test as per ASTM C143/C143M-20 [52]. The initial and final setting time of the alkali-activated slag-based paste was determined by penetration resistance measurements using a 1 mm Vicat needle according to ASTM C191-19 [53]. The compressive strength of AASC mixes was measured according to BS1881:Part116 [54]. The tests were performed on 100 mm cubic specimens per mixture at 7 and 28 days using a compressive testing machine with a maximum capacity of 200 Ton.

#### 3.3.2. Physical and Mechanical Properties of FR-AASC

**Figure 2:**
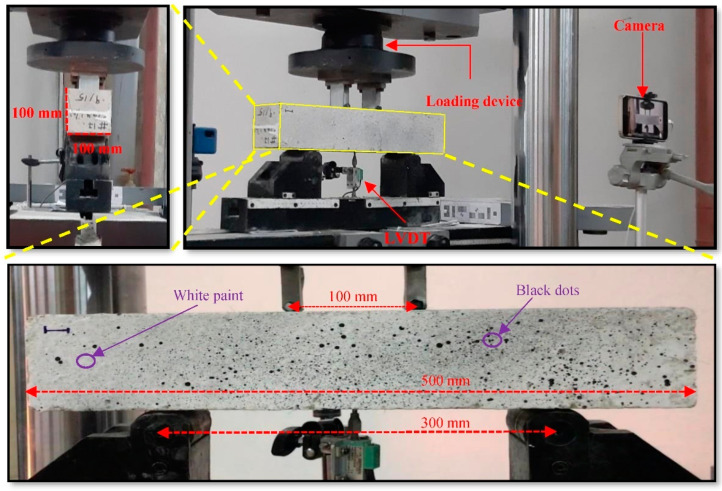
Four-point bending test setup for flexural strength determination and crack evolution assessment through the DIC technique.

The workability of FR-AASC was also determined as per ASTM C143/C143M-20 [52] to compare with non-fibrous AASC. The specific density of fresh FR-AASC was calculated by dividing the net mass of the mixture by the volume of the measure in which the mixture was placed, according to ASTM C138/C138M-17a [55]. The 28-day cubic specimens for each FR-AASC mixture were considered to calculate 3-day water absorption (W) using Equation (1), as per ASTM C642-13 [56].

(1) $$W=\frac{M_{b}-M_{a}}{M_{a}}$$

where M_a_ presents the mass of the oven-dried sample in the air (g), and M_b_ shows the mass of the surface-dry sample in the air after three days of immersion in water (g). To measure the 28-day compressive strength of FR-AASC, cylindrical specimens with 100 mm diameter and 200 mm height were fabricated and tested for each mixture based on ASTM C39/C39M-21 [57]. The axial loading was applied at 0.01 mm/s by a servo-controlled device. The flexural behavior of the mixtures was studied by conducting four-point bending tests, based on ASTM C1609/C1609M-19a [58], on 28-day prismatic samples per mixture. A servo-controlled device was used to apply displacement to the specimen at the constant rate of 0.008 mm/s. In order to record the vertical displacement in the mid-span of the prismatic beam, an LVDT was situated there. The setup of the bending test and specimen geometry is shown in Figure 2.

A non-contact method of deformation measurement, i.e., 2D digital image correlation (DIC), was employed to investigate how the cracks appeared and developed. Moreover, gradual changes the strain field underwent could be effectively tracked by this technique. Before testing, the specimens were primarily prepared to obtain appropriate images for analysis. In this regard, the prismatic beams were painted with white color, and then random black dots were sprayed on their lateral surface. A high-resolution camera was fixed on the ground to avoid any movement. Taking images and the loading process were both started simultaneously. The experiment was conducted during the day to ensure sufficient surrounding light (Figure 2). The images (capturing one image per 5 s) were then processed by Ncorr software to attain strain distribution and assess crack evolution.

**Figure 3:**
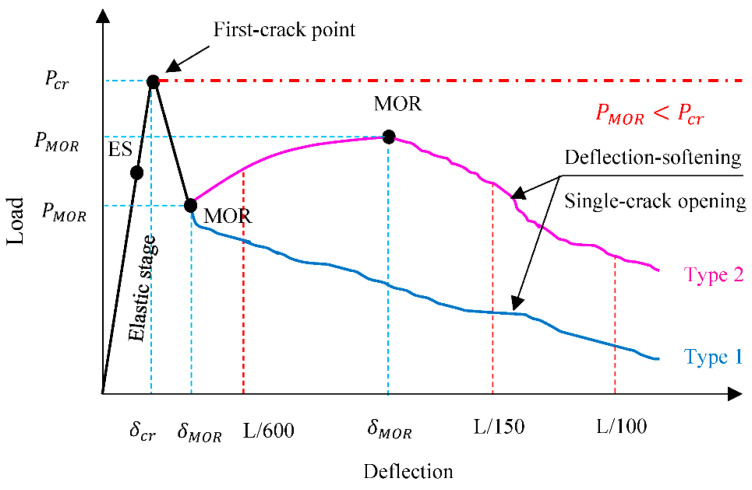
Typical behavior of deflection-softening materials under bending test.

In order to analyze the performance of FR-AASC in bending, several indices were utilized in this study, which are indicated in Figure 3. According to ASTM C1609/C1609M-19a [58], the first zero slope point on the load-deflection curve is recognized as the first-crack point. The first-crack strength can be obtained by inserting the first-crack load in Equation (2).

(2) $$f_{\mathrm{cr}}=\frac{P_{\mathrm{cr}}L}{{\mathrm{bd}}^{2}}$$

where f_cr_ and P_cr_ are the first-crack strength (MPa) and load (kN), respectively, L is the length of span (300 mm), b is the width (100 mm), and d is the height (100 mm) of the cross-section. The f_MOR_ is the strength at the point called the Modulus of Rupture where softening starts to occur after the first-crack point. The f_L/600_, f_L/150_, and f_L/100_ are the strength at the equivalent deflection level, regarding the L as 300 mm. The flexural toughness T_cr_, T_L/600_, T_MOR_, T_L/150_, and T_L/100_ are defined as the measured area beneath the load-deflection curve up to the prescribed points. The L/100 deflection level is the additional point, recommended in [59], to completely distinguish the behavior between mixtures with various fiber dosages. Toughness indices (I_5_, I_10_, and I_20_), and residual strength factors (R_5,10_ and R_10,20_) are also derived from the load-deflection curve, in accordance with ASTM C1018-97 [60].

The classification of the flexural behavior of fiber-reinforced concrete is generally performed with respect to the post-cracking load-carrying capacity. The FR-AASC is classified as a deflection-hardening material if it sustains loads of a higher degree than that of the first crack. Otherwise, it is known as deflection softening. Mixtures reinforced with low fiber volume exhibit deflection-softening behavior [61]. Two types of deflection-softening responses are illustrated in Figure 3. In type 1, after the appearance of the first crack at the endpoint of linearity (LOP), a sudden drop of load occurs, and the load continues to decline by increasing the deflection. In contrast, in type 2, there is still a rise in the applied load after the first-crack emergence, although it cannot overcome the first-cracking load.

### 3.4. Optimization Method

In this research, the Taguchi method was used as an optimization technique to design the experiment. After that, the Grey relational analysis was merged with the Taguchi method to achieve multiple optimizations of performances. The concept of Analysis of variance (ANOVA) analysis was utilized to investigate the importance order of factors. Finally, the confirmation experiment was performed to evaluate the reliability of the Taguchi-Grey method for AASC design.

#### 3.4.1. Determination of Parameters and Responses

Five main parameters, which might influence the fresh and hardened characteristics of AASC, were considered as experimental factors. The selected parameters were the binder content, the molarity of sodium hydroxide (SH) solution, the alkaline solution to binder content (Al/Bi) ratio, the water to alkali-activated solids (W/S) ratio, and the sodium silicate to sodium hydroxide solutions (SS/SH) ratio, labeled by A, B, C, D, and E, respectively. Each parameter had four levels, as plotted in Table 2. In the W/S ratio parameter, the total mass of water (W) was the sum of water content within the SS and SH solutions and the mass of extra water. Meanwhile, the total alkali-activated solids content (S) was the sum of the mass of slag and solid substances contained in the alkaline solution (SH and SS). On the other hand, since the rapid setting time and poor workability of AASC limited its actual use in the structural application, five responses of the workability, setting time (initial and final), and compressive strength (7 and 28 days) were selected as quality responses of AASC in this study.

#### 3.4.2. Selection of Taguchi Orthogonal Array

The Taguchi method developed the orthogonal array as a set of matrixes to reduce the total number of experiments. The appropriate orthogonal array can be chosen based on the total degrees of freedom (DOF). It means that the orthogonal array’s DOF should not be smaller than the total DOF [62], which is the sum of the singular degree of freedom of each factor calculated in Equation (3) [20].

(3) $${\mathrm{DOF}\;}_{\mathrm{factor}}=L-1$$

where L is the number of levels. The total degree of freedom was equivalent to fifteen as each of the five parameters had four levels (DOF of 3). So, the Taguchi L_16_ orthogonal array could be utilized because it has the same DOF (15) as the total DOF. Additionally, the minimum number of experiments (N) that must be performed was computed as 16 based on Equation (4) [13].

(4) $$N=1+(L-1)\times P_{d}$$

where P_d_ shows the number of design parameters. Therefore, an L_16_ orthogonal array, in which only 16 experiments are required to estimate the optimized mixture, can be an excellent alternative to all possible 1024 (4^5^) experimental mixtures presented by full factorial design. The experimental runs as per the L_16_ orthogonal array are illustrated in Table 3.

#### 3.4.3. Signal-to-Noise Ratio

A statistical measure of performance known as the signal-to-noise (S/N) ratio is advocated by the Taguchi method to minimize the influence of uncontrollable (noise) factors [63]. This single indicator determines the relative importance of the selected parameters by considering the average value and standard deviation of obtained results jointly and simultaneously [19]. Depending on the desired objective characteristics, three types of S/N ratio analysis can be employed: the higher-the-better (HB), the lower-the-better (LB), and the nominal-the-best (NB). The current study aims to maximize all characteristics of AASC concrete. In other words, higher compressive strength and workability and, at the same time, longer setting times were selected as goals. Thus, the S/N ratio was computed based on the HB concept according to Equation (5) [64].

(5) $$S/N=-10\times \log_{10}\left(\frac{1}{n}\sum_{i=1}^{n}\frac{1}{Y_{i}^{2}}\right)$$

where the number of mixtures is given by n, and Y_i_ represents the experimental data of the i^th^ mixture.

#### 3.4.4. Grey Relational Analysis

Grey relational analysis (GRA), which can convert the multi-response function into a single-response function, was employed to achieve the best performance in terms of the compressive strength (7 and 28 days), setting time (initial and final), and workability of AASC simultaneously. Data processing, performed based on the GRA method, is stated as follows.

- Grey relational generating;

This math operation, which converts the original sequences into comparable sequences (termed as grey relational generating), is intended to decrease variability and avoid the effect of using various units [65,66]. Since the expectancy is the maximization of all responses in the current study, all trial runs data of the S/N ratio were normalized by employing Equation (6) [67] based on the HB condition.

(6) $$Z_{\mathrm{ij}}=\frac{Y_{\mathrm{ij}}-\min\left(Y_{\mathrm{ij}}\right)}{\max\left(Y_{\mathrm{ij}}\right)-\min\left(Y_{\mathrm{ij}}\right)}$$

where Z_ij_ is the normalized S/N ratio of the i^th^ mixture for the j^th^ response, and Y_ij_ denotes the S/N ratio of the i^th^ mixture for the j^th^ response.

- Grey relational coefficient;

To show the relation among the desired (ideal) and actual normalized experimental data, the grey relational coefficient (GRC) was calculated based on normalized S/N ratio data using the following Equation (7) [68].

(7) $${\mathrm{GRC}}_{\mathrm{ij}}=\frac{\Delta_{\min}+\psi \Delta_{\max}}{\Delta_{\mathrm{ij}}+\psi \Delta_{\max}}$$

where GRC_ij_ presents the grey relational coefficient of the i^th^ experiment for the j^th^ response and ψ is the distinguished coefficient that is usually taken as 0.5 [19]. ∆_ij_ represents the deviation sequence which is the absolute value of the difference between the ideal value of the normalized S/N ratio and the normalized S/N ratio [20,66]. ∆_min_ and ∆_max_ are the minimum and maximum values of the deviation sequence, respectively.

- Grey relational grade;

The gray relational grade (GRG), representing the level of the correlation between the referential sequence and comparative sequence [69], was computed by averaging the GRC that corresponds to the chosen responses based on the following Equation (8) [69].

(8) $${\mathrm{GRG}}_{i}=\frac{1}{n}\sum_{j=1}^{n}W_{j}{\mathrm{GRC}}_{\mathrm{ij}}$$

where GRG_i_ denotes the grey relational grade of the i^th^ experiment, n is the number of performance characteristics (responses), and W_j_ represents the weight factor assigned to the j^th^ response. In this paper, as the importance of properties of AASC (responses) was the same, equal weight was considered for all responses.

- Optimal levels of factors;

The optimum level of each examined parameter can be determined based on the GRG. Since the GRG presents the degree of correlation among the reference and the comparability sequences, the optimal levels can be measured from the highest GRG of each parameter. So, for the experimental parameter (i), the optimum level (j^∗^) was determined based on Equation (9) [67].

(9) $$j^{*}=\max\left(\zeta_{\mathrm{ij}}\right)$$

where $\zeta_{\mathrm{ij}}$ is the mean of the GRG level j of parameter i. The importance order of parameters can also be established according to GRG. The effect of each experimental parameter was calculated based on the difference in mean GRG between high and low levels, as presented in Equation (10) [67].

(10) $$\Delta_{i}=\max\left(\zeta_{\mathrm{ij}}\right)-\min\left(\zeta_{\mathrm{ij}}\right)$$

where $\Delta_{i}$ is the effect of parameter i.

#### 3.4.5. Analysis of Variance

Analysis of variance (ANOVA), proposed by Ronald Fischer as a statistical technique, was employed to evaluate the contribution of each variable in the experiment. The ratio of the mean of squared deviations to the mean of squared errors ratio (F-value) shows the relative significance of each parameter of the experiment. In this research, since the degree of freedom of error was zero due to the selection of an over-fitted design [13], the contribution of each factor on the properties of AASC (responses) was calculated based on Equation (11) [70].

(11) $$\mathrm{contribution}=\frac{\mathrm{sum}\;\mathrm{of}\;\mathrm{square}\;\mathrm{of}\;\mathrm{variable}}{\mathrm{total}\;\mathrm{sum}\;\mathrm{of}\;\mathrm{squares}}\times 100$$

#### 3.4.6. Verification Experiments

The confirmation process was carried out to investigate the accuracy and applicability of the proposed method. The optimal value of the GRG can be predicted based on Equation (12) [68].

(12) $$Y_{e}=Y_{m}+\sum_{i=1}^{p}\left(\bar{Y_{i}}-Y_{m}\right)$$

where Y_e_ is the optimal value of the GRG, Y_m_ represents the total average of the GRG, $\bar{Y_{i}}$ is the average of the GRG value at the optimal level for the i^th^ parameter, and p shows the number of examined parameters.

## 4. Results and Discussion

### 4.1. Optimization of AASC

Based on the L_16_ orthogonal array presented by the Taguchi method, the experimental runs were carried out, and the responses corresponding to different variables were recorded. The obtained results and computed S/N ratio are illustrated in Table 4. The Grey Relational Analysis based on procedures explained in Section 3.4.4 was performed on the results of L_16_ orthogonal arrays. The results of the normalized data, deviation sequences, grey relational coefficients (GRC), grey relational grade (GRG), and grey relational order (rank) are illustrated in Table 5.

Among all sixteen cases, the highest GRG (0.83) was attained for TM10. The higher the GRG value is, the closer it is to the ideal value [66,68]. In other words, an experiment with the highest grey relational grade gives the best multiple output responses. Therefore, experimental trial 10 provided the maximum multiple quality characteristics among the L_16_ orthogonal array.

From Table 5, it is also evident that the slump response obtained the highest mean GRC of the reference sequences, indicating that the slump had the strongest reference sequence as compared to other responses. A higher mean GRC implies a stronger correlation to parameters [71]. So, workability has the highest correlation to examined parameters than others and can be easily affected by selected factors. The average of the GRG for every level of the examined parameters to obtain the optimum combination of parameters was measured and illustrated in Table 6.

The optimum level of each experimental parameter (highest mean of GRG) is denoted in bold. Thus, the best combination for optimum AASC parameters was a binder content of 400 kg/m^3^, 14 M of sodium hydroxide, an AL/B ratio of 0.55, a W/S ratio of 0.4, and an SS/SH ratio of 1.75. In other words, the A3B3C4D3E1 mixture was the optimal one of all possible mixtures. As can be seen from the main effect table (Table 6), the Al/Bi ratio was the most substantial parameter in response to owning the highest delta value, indicating that it has the strongest correlation to responses [71]. The second most important factor was the binder content, followed by SH concentration, SS/SH ratio, and W/S ratio.

The analysis of variance was also performed on grey relational grades to identify the contribution percentage of individual input parameters on multiple output responses. From Table 7, which illustrates the ANOVA results, it can be noticed that the Al/Bi ratio was the most influential factor on multi-performances of AASC with a percentage of participation of more than 50%, followed by binder content and SH concentration with almost the same influence of approximately 17% on the multi-responses. The impact of SS/SH and W/S on the experiments was also found insignificant compared to other controlled factors. The result from ANOVA is in concordance with the main effect table (Table 6) for means of GRG.

In the final step, the verification experiment was conducted to compare the performance of the A3B2C4D3E1 (TM10) mixture, which was considered as the initial optimum mix with the highest GRG value between 16 mixtures proposed by Taguchi, and A3B3C4D3E1 (named as TM17), which was established as the optimal mixture based on the mean of GRG. So, by using the same procedures as previous runs, the verification tests were conducted on TM17. After that, the experimental value of the GRG of TM17 corresponding to the recorded results of confirmation tests was calculated. Additionally, the predicted optimal value of the GRG was computed utilizing Equation (12). The data of the confirmation experiment is illustrated in Table 8.

From Table 8, it is evident that the predicted value of the GRG agreed well with the experimental value. The GRG of the optimized mix (TM17) was the highest among all sixteen previous trial mixes (TM1–TM16), indicating that using the combination of optimal parameters proposed by the Taguchi-Grey relational analysis resulted in improvement in multiple properties of AASC.

### 4.2. Physical and Mechanical Properties of FR-AASC

The effect of RTSF incorporation on the physical and mechanical properties of AASC was assessed in this section. To do so, the optimized AASC mix (A3B3C4D3E1), proposed in Section 4.1, was considered as the control mix design, and RTSF was added to the mixture, as shown in Table 9.

#### 4.2.1. Workability

As can be seen in Table 9, RTSF incorporation had a negative effect on workability. When the fiber volume fraction increased from 0% to 0.6%, the slump value decreased by around 54% from 195 mm to 90 mm. This can be attributed to the shear resistance provided by the random distribution of RTSF, which resulted in the creation of a skeleton to free flow [72]. Furthermore, the deformed fibers could increase the anchorage between fibers and aggregates [1]. Meanwhile, different shapes of fibers led to the formation of a network with each other inside the matrix, which consequently contributed to a growth in the yield stress of fresh concrete [73,74]. Thus, the higher the fiber content was, the lower the workability would be.

#### 4.2.2. Demolded Density and Water Absorption

**Figure 4:**
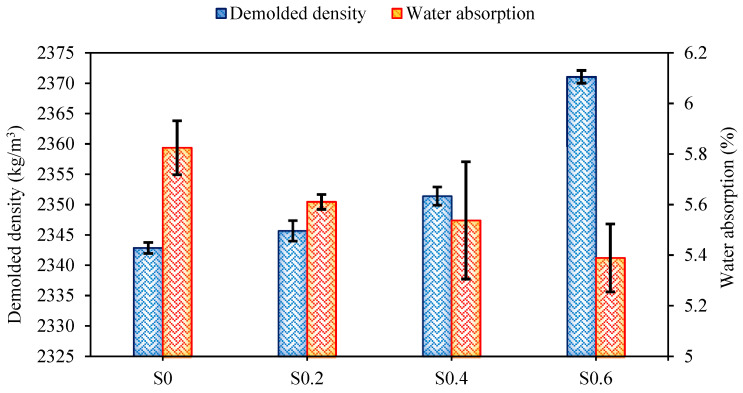
Demolded density and water absorption of the FR-AASCs.

Figure 4 illustrates the recorded results in terms of demolded density and water absorption of plain and fiber-reinforced mixes.

The density of the control mix (plain) was calculated at 2343 kg/m^3^. With the incorporation of 0.2%, 0.4%, and 0.6% RTSF by volume fraction in the AASC mixture, the density increased to 2346, 2351, and 2371 kg/m^3^, respectively. The increase in demolded density can be ascribed to the higher specific density of RTSF compared to the AASC matrix [75,76]. As can be seen in Figure 4, when the volume fraction of RTSF increased from 0% to 0.6%, the water absorption slightly diminished from 5.65% to 5.25%. This reduction could be explained by the random distribution of short fibers, which prevented the development of the micro-cracks by stitching them [77,78] and reducing the number of voids by the occupation of pores [79].

#### 4.2.3. Compressive Strength

**Figure 5:**
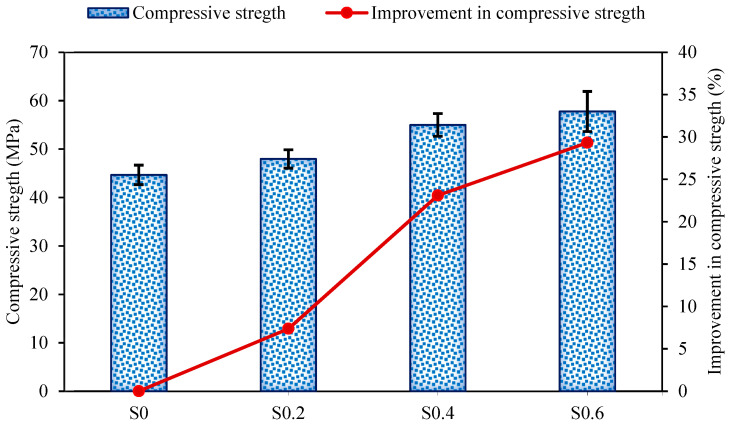
Compressive strength of FR-AASCs.

The compressive strength development of AASC reinforced with RTSF regarding different volume fractions is illustrated in Figure 5. As can be seen, the unreinforced AASC (S0) possessed the compressive strength of 44.67 MPa, which was improved by 7.4%, 23.1%, and 29.3% by the addition of 0.2%, 0.4%, and 0.6% RTSF to the AASC mixture, respectively. This increase could be ascribed to the hydrophilic nature of RTSF, causing a strong fiber-matrix bond [76]. Additionally, increasing the RTSF content resulted in smaller fiber spacing. Therefore, more fibers are available to bear the load, leading to the multi-cracks. Meanwhile, stiff RTSF could retard the crack extension, which consequently led to an improvement in the compressive strength [37,38,72,74].

**Figure 6:**
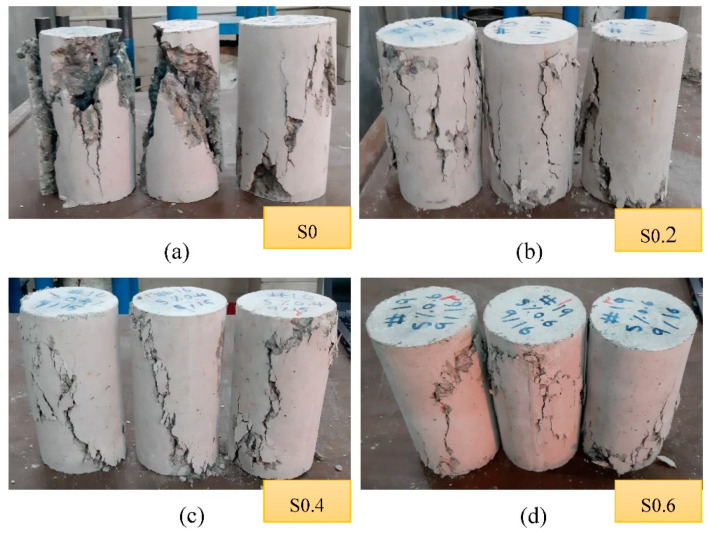
Failure patterns observed in compressive strength test (**a**) S0 (**b**) S0.2 (**c**) S0.4 (**d**) S0.6.

The crack patterns that occurred in the unreinforced and RTSF-reinforced cylindrical specimens are illustrated in Figure 6. The presence of RTSF in AASC mixtures drastically transformed the failure mode in compressive specimens. The specimens containing no RTSF, S0, showed a brittle failure mode with a well-formed cone on one end, and vertical cracks extended through another end. After attaining ultimate compressive strength, S0 split into segments suddenly and loudly. Aside from developing compressive strength, RTSF could efficiently alter the failure pattern to a more ductile one. Thanks to the bridging effect of RTSF, reinforced specimens survived intact until the failure point. Multiple vertical cracks toward the ends of the cylinder with no formed cone were visualized for the S0.2 specimen. However, by increasing the RTSF volume fraction to 0.4% and 0.6%, cylindrical specimens were more likely to fracture in a diagonal pattern. This implies that the higher RTSF content provided a higher confining effect, which prevented severe columnar vertical cracks.

#### 4.2.4. Flexural Performance of FR-AASC

##### Load-Deflection Behavior

**Figure 7:**
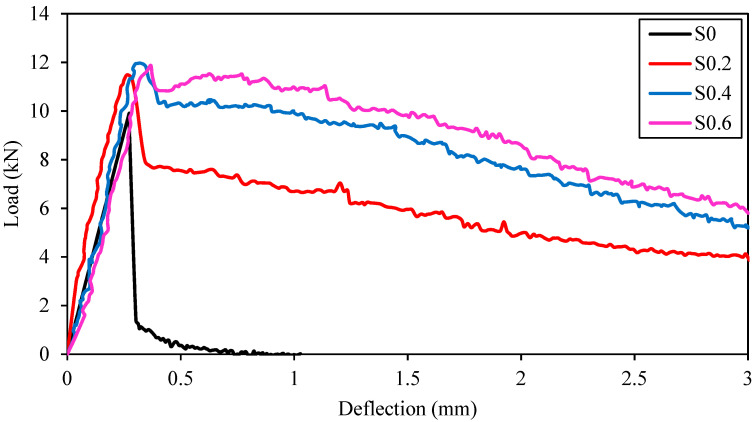
Load-deflection curves of alkali-activated mixtures containing various RTSF fractions subjected to four-point bending tests.

The load-deflection curves of alkali-activated mixtures containing various RTSF fractions are depicted in Figure 7. The unreinforced specimen (S0) behaved brittle and experienced a rapid degradation of load after cracking owing to the fiber shortage. By RTSF inclusion, the load-bearing capacity of FR-AASC mixtures was improved, and the manner of fracture became ductile. All FR-AASCs experienced an immediate drop in load when they cracked. The higher drop on the load-deflection curve represented a larger crack width [38]. Increasing the RTSF volume fraction caused a smaller drop in the load. This behavior stemmed from the irregular geometry of stiff RTSF, which became damaged during the recycling process (i.e., shredding process) [80]. Additionally, utilizing slag as the binder in alkali-activated concrete turned the smooth surface of steel fibers to coarse [24]. Therefore, the strong friction between RTSF and matrix, provided by a higher number of longer fibers, could limit the stress drop and, as a result, restrict the crack width [1,38].

Considering the post-cracking stage, a transformation in the failure mode from type 1 into type 2 was observed by the inclusion of a higher fiber dosage. A slight deflection-hardening stage started to appear as RTSF content increased due to a growth in the bonding force between the fibers and matrix [1]. Besides, this light deflection-hardening phenomenon was rooted in the enhanced efficiency of fiber reinforcement in the AASC mixture, which was due to the early activation of fibers in micro-cracks formed by shrinkage before applying the mechanical loading [23]. The rise in the fiber volume fraction resulted in the steeper gradient of the descending branch of the load-deflection curve and reduced ductility due to the intrinsic brittle nature of the high-modulus fiber, which is in line with the findings of a previous study [37].

##### Equivalent Bending Strength (Load Carrying Capacity)

The first-crack strength (f_cr_) value of the unreinforced specimen, S0, was 2.96 MPa. Regardless of the content, RTSF addition caused an increase in the f_cr_ value by approximately 19.3%, in comparison with S0, and reached the average value of 3.53 MPa. Micro RTSFs were able to bridge the micro-cracks and prevent or delay their localization. Therefore, the elastic stage was lengthened, and a greater cracking strength was achieved [1,81]. First-crack deflection (δ_cr_) ranged from 0.27 mm in S0 to 0.37 mm in S0.6 in the elastic stage, indicating that the presence of RTSF did not have a considerable effect on δ_cr_ due to the fact that the first crack was mainly influenced by matrix strength rather than fiber bridging [1,37,82].

**Figure 8:**
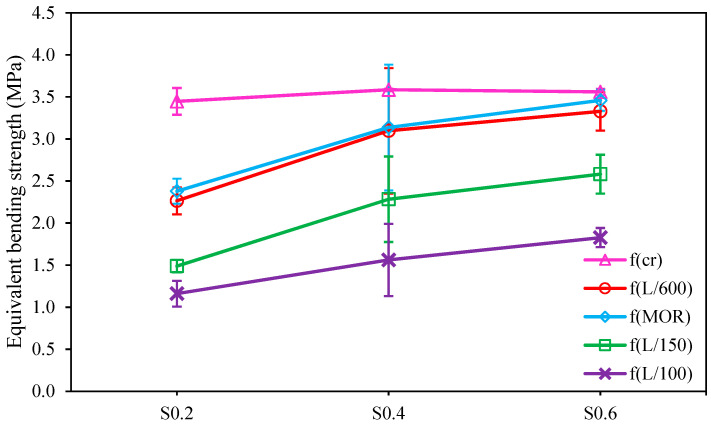
Impact of RTSF dosage on the equivalent bending strength of FR-AASCs.

Figure 8 is presented to assess the equivalent bending strength of FR-AASCs at prescribed points. The impact of RTSF dosage on f_cr_ was not significant while increasing RTSF content caused a growth in the strength of the post-cracking stage. S0.6 attained the highest Modulus of Rupture (f_MOR_) of 3.46 MPa, which was around 10.3% and 45.4% higher than that of S0.4 and S0.2, respectively, implying that fibers played a major part in restraining the crack propagation across the failure surface through fiber bridging action [24,37]. Considering the post-peak performance, all FR-AASCs were in the deflection-softening stage at the L/150 and L/100 deflection points, meaning that the ductility of the unreinforced mixture was improved by fiber inclusion. A trend similar to what happened at the MOR point was observed at the L/150 and L/100 deflection points; that is, the equivalent bending strength was improved by increasing the RTSF content. In terms of residual strength, S0.6 performed the best, while S0.2 performed the worst, as can be seen in Figure 8.

##### Flexural Toughness

**Figure 9:**
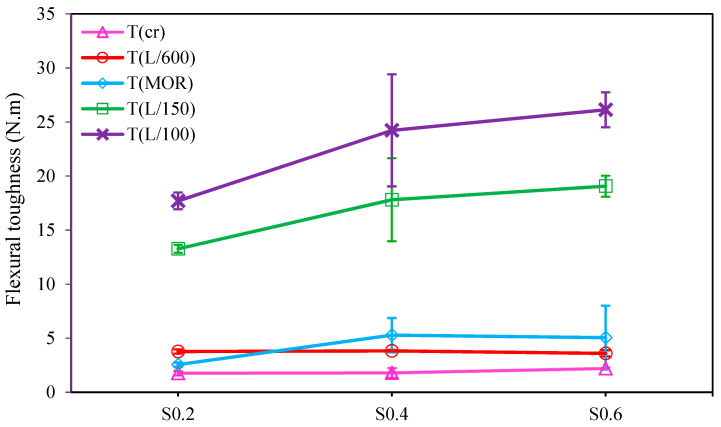
Impact of RTSF dosage on the flexural toughness of FR-AASCs.

In order to minimize the hazards related to sudden brittle failure, providing materials with sufficient energy absorption capacity seems to be quite essential. The flexural toughness of FR-AASCs at prescribed points is depicted in Figure 9. As indicated, despite increasing the RTSF volume fraction, the flexural toughness value at the first-crack point (T_cr_) remained almost constant. A similar trend was also observed at the L/600 and MOR points. In such low deflection values (i.e., δ_cr_, L/600, and δ_MOR_), the fiber content did not significantly affect flexural toughness, and the cracking behavior was mainly governed by matrix strength instead of fiber bridging [82]. It is worth noting that S0.2 started to soften after cracking and was in the deflection-softening stage at the L/600 deflection point, leading to a higher toughness at L/600 compared to MOR and lower flexural toughness at MOR in comparison with other mixtures. By increasing the deflection value from δ_MOR_ to L/150, the difference between the flexural toughness values of all FR-AASC mixtures became larger, meaning that fibers bridged the crack after MOR to slow down its opening rate. Additionally, by increasing the volume fraction of RTSF in AASC mixtures, the number of macro steel fibers increased so that more crack bridging effect was provided, and higher flexural toughness was achieved. This is consistent with the results obtained in [37,82], that higher fiber content in concrete can bring about a larger amount of energy dissipation due to the fiber bridging action. S0.6 possessed the highest T_L/100_ (26.12 N·m) while the lowest T_L/100_ belonged to S0.2 (17.70 N·m). Therefore, S0.6, in general, depicted the highest energy absorption capacity.

**Figure 10:**
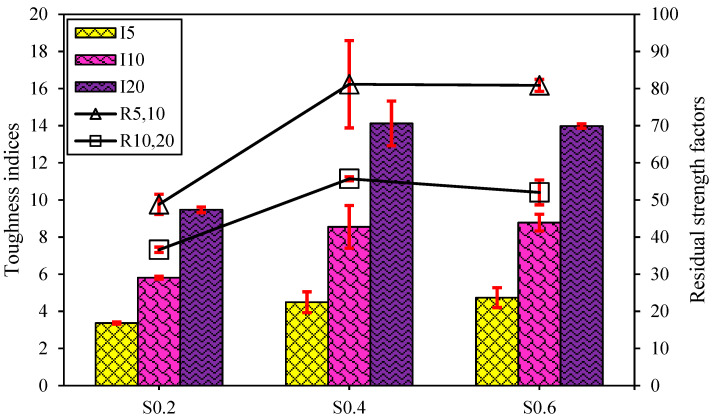
Impact of RTSF dosage on the toughness indices and residual strength factors of FR-AASCs.

**Figure 11:**
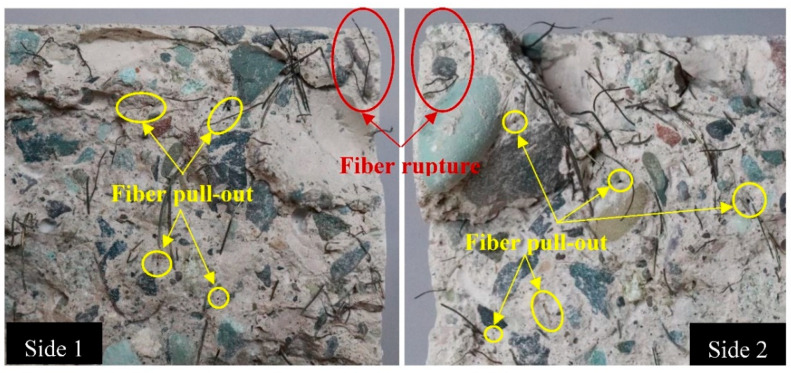
Both sides of the failure surface of the flexural specimen.

To investigate the energy absorption capability after cracking, the toughness indices for all FR-AASC mixtures were calculated and displayed in Figure 10. As shown, the S0.2 specimen exhibited the lowest performance with I_5_, I_10_, and I_20_ values of 3.37, 5.81, and 9.47, respectively, which were around 26.9%, 32.9%, and 32.6% lower than those of the other two mixtures, respectively. The toughness indices of the S0.4 and S0.6 specimens were so close to each other. For these mixtures I_5_, I_10_, and I_20_ were calculated at approximately 4.61, 8.66, and 14.05, respectively. Increasing the RTSF volume fraction from 0.2% to 0.4% was accompanied by a growth in post-cracking toughness. The positive influence of RTSF on the toughness of FR-AASC was rooted in the hydrophilic surface of steel fibers, causing a strong bond with the alkali-activated matrix. Therefore, FR-AASC incorporating a higher dosage of RTSF could dissipate more energy before the fiber-matrix bond failure [37,38,76]. However, further increasing the RTSF content to 0.6% had no significant effect on the toughness indices. This could be related to the increased RTSF dosage in the AASC mixtures leading to the inappropriate dispersion and balling of them. Additionally, the inconsistent behavior of each RTSF due to the irregular shape and damaged surface after the recycling process resulted in obtaining FR-AASC samples with uncertain properties and high deviation [83,84]. Finally, owing to the high bonding strength of irregular RTSF with matrix, the fiber rupture might occur during the fiber pull-out process [85]. Since fiber pull-out was the most favorable pattern of fiber failure due to the high frictional forces with the matrix, which needed to be overcome [86], fiber rupture led to the lower area under the load-deflection curve and, consequently, decreased toughness characteristics [83]. Figure 11 exhibits the failure surface of an RTSF-reinforced specimen under flexural loading.

The impact of fibers on the residual strength factors of FR-AASC mixtures, as the significant signs of ductility [83], is also illustrated in Figure 10. A trend similar to that observed for toughness indices was noticed in the case of residual strength factors by the rising RTSF volume fraction from 0.2% to 0.6%. The S0.4 mixture outperformed all other series with the highest R_5,10_ and R_10,20_ of approximately 81.16 and 55.70, respectively, slightly higher than those of S0.6.

##### Analysis of Crack Propagation Utilizing the DIC Technique

**Figure 12:**
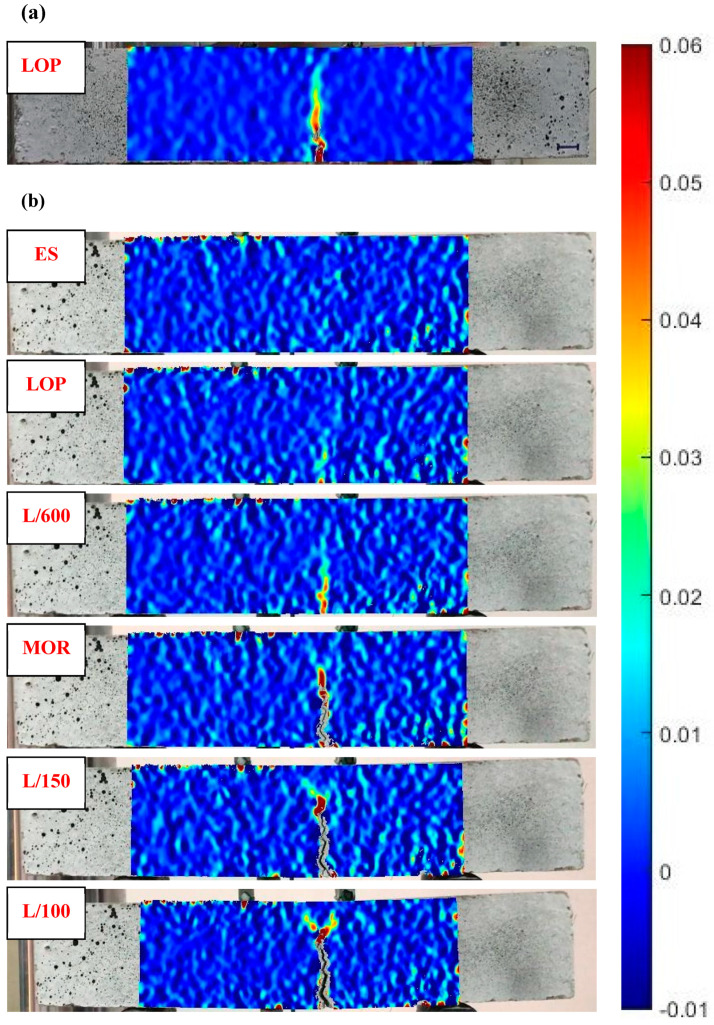
DIC images indicating crack propagation in bending specimens at key points shown in Figure 3. (**a**) S0 specimen. (**b**) S0.6 specimen.

The DIC method was adopted to thoroughly discuss the crack evolution in prismatic beams during flexural loading. To reasonably contrast the cracking behavior of various mixtures, the DIC images were obtained by the same scale and color, and the region of interest was cropped [87]. As shown in Figure 12a, the DIC image of unreinforced AASC (S0) at the first-crack point (LOP) represented obvious red and yellow colors, indicating the sudden crack occurrence with one tip. Figure 12b displays the DIC images corresponding to the flexural failure of the S0.6 specimen. There was no evidence of cracking in the elastic stage (Figure 12b, ES), and the strain was evenly distributed with a low value. As opposed to the S0 specimen with a clear concentration in the cracking tip, no crack tip was observed in the first-crack point of S0.6 (Figure 12b, LOP), and slightly increased strain (i.e., the yellow and orange colored area) was related to the hairline crack initiation. This suggested that RTSF contributed to the well-dispersion of stress and restraining the advancing crack through crack-bridging action at the micro level [30,87]. Once the MOR point was attained (Figure 12b, MOR), the main crack propagated towards the middle height of the sample. A further increase in load in the softening stage (Figure 12b, L/150 and L/100) resulted in a twisty crack with multiple branches at the crack tip owing to the fiber-bridging effect [88] and, as a result, enhanced the effective cracking path. Increasing the flexural load also caused the crack to widen.

**Figure 13:**
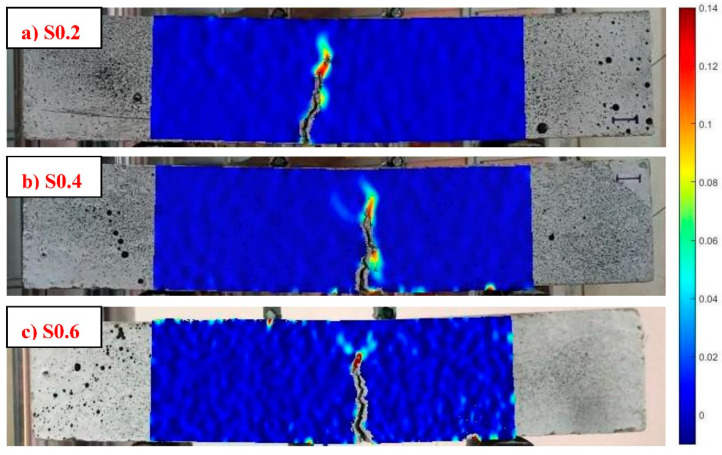
DIC images corresponding to the L/100 (3 mm) deflection level.

**Figure 14:**
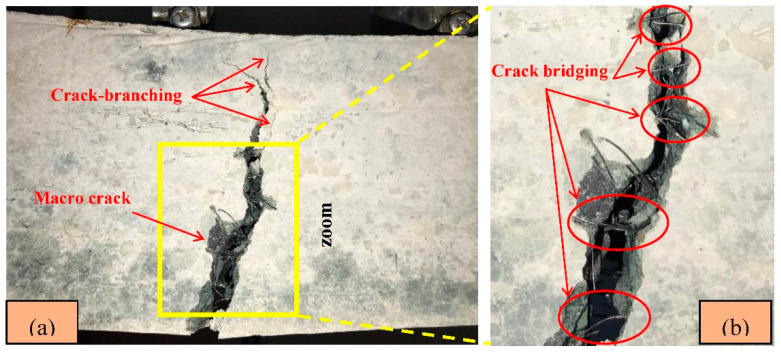
Failure pattern of an RTSF reinforced specimen.

The DIC images corresponding to the L/100 (3 mm) deflection level of all FR-AASC mixtures are demonstrated in Figure 13. The formation of a twisty main crack in fibrous mixtures revealed that the failure mode underwent a transformation from the brittle mode in S0 to a ductile one in reinforced mixtures due to the fiber-bridging action [83,88]. The crack-branching phenomenon alongside and at the tip of the main macro-crack was observed by increasing the RTSF volume fraction, as indicated in Figure 14a. This could be ascribed to the increased number of micro RTSF, which comprised fibers with varying lengths, and the smaller fiber spacing. Therefore, more fibers were available in the vicinity of the main macro-crack to bridge the micro-cracks, avoid the opening at the tip of the main macro crack, postpone its development, and enhance the load and deformation capacity in the softening stage. Macro fibers played a major part in bridging macro-cracks (Figure 14b) and imposing ductility on the mixture due to the longer length [86]. Thus, improved strength and energy absorption capacity could be provided by the higher number of macro-fibers.

## 5. Conclusions

The Taguchi-Grey relational analysis was performed to obtain the optimal combination of the considered parameters (binder content, the molarity of sodium hydroxide solution (SH), the alkaline solution to binder content (Al/Bi) ratio, the water to alkali-activated solids (W/S) ratio, and the sodium silicate to sodium hydroxide (SS/SH) solution ratio) for alkali-activated slag-based concrete (AASC) with the consideration of multiple performance characteristics (workability, setting time, and compressive strength). Then, the influence of adding recycled tire steel fiber (RTSF) as a reinforcing material on the physical (workability, demolded density, and water absorption) and mechanical (compressive strength and flexural behavior) properties of optimized AASC was investigated. Based on the results reported in this study, the following conclusions can be highlighted:

1. Based on the results of grey relation analysis, the optimal AASC mixture in terms of multi-response is obtained from the A3B3C4D3E1 combination with a binder content of 400 kg/m^3^, SH molarity of 14 M, Al/Bi ratio of 0.55, W/S ratio of 0.40, and SS/SH ratio of 1.75. The Al/Bi ratio and workability have the strongest correlation to responses and parameters, respectively.
2. The results of the ANOVA of the GRG showed that the Al/Bi ratio has the greatest influence (57%) on multiple responses of AASC, followed by the binder content (18%), the molarity of SH (16%), the SS/SH ratio (8%), and the W/S ratio (1%).
3. Based on the confirmation tests results, the overall performance characteristics of AASC are improved, and the highest GRG among all AASC mixes is attained, indicating that the combination of optimal parameters proposed by Taguchi-Grey relational analysis performs well in terms of multiple characteristics of AASC.
4. Considering the physical properties, the inclusion of RTSF in the AASC mixtures causes a reduction of 54% in workability, a slight rise of 1.2% in demolded density, and a slight reduction of 7.5% in water absorption when the RTSF volume fraction increases from 0% to 0.6%.
5. The compressive strength of AASC improves by nearly 30% with the inclusion of a 0.6% volume fraction of RTSF (S0.6) and reaches 57.8 MPa. The vertical columnar cracking pattern in unreinforced cylindrical specimens (used for compressive strength tests) changes to distributed small vertical cracks and diagonal cracking patterns as the RTSF dosage in the mixture increases.
6. Regarding flexural behavior, RTSF incorporation in the AASC mixture transforms the brittle mode of failure to ductile. A slight deflection-hardening stage in the load-deflection curve of bending specimens emerges when the RTSF content increases. A growth of 19.3% in first-crack strength (f_cr_) is recognized by increasing the RTSF content, while it seems to have no significant influence on first-crack deflection (δ_cr_). Accordingly, the post-cracking performance of fiber-reinforced AASC (FR-AASC) in terms of strength enhances via the fiber-bridging mechanism. S0.6 attained the highest Modulus of Rupture (f_MOR_) of 3.46 MPa, about 10.3% and 45.4% higher than that of S0.4 and S0.2 with 0.4% and 0.2% volume fractions of RTSF, respectively.
7. The outstanding contribution of fiber bridging to the flexural toughness becomes evident at high deflection values. In this regard, the more the fiber content, the higher the flexural toughness. For example, S0.6 attained the highest toughness of 26.12 N. m at a mid-span deflection of 3 mm (T_L/100_), whereas S0.2 had the lowest T_L/100_ (17.70 N. m).
8. Due to the balling of fibers with a 0.6% volume fraction in the AASC mixture, the inconsistent properties of RTSF and fiber rupture, toughness indices, and residual strength factors level off in 0.4% RTSF volume fraction after growth is observed due to increasing the fiber content from 0.2% to 0.4%.
9. With the help of the DIC technique in tracing the crack evolution, it is observed that increasing the RTSF content postpones crack development by bridging action. Additionally, a single crack is transformed into a twisty branched crack due to the shorter distance between the fibers of the high-volume fraction.

This study demonstrated the practicability of Taguchi-Grey relational analysis in the design of AASC and took a step forward by investigating the effects of RTSF inclusion on the engineering properties of FR-AASC. For further investigation, the RTSF should be incorporated into the Taguchi-Grey analysis and the quantity of fibers could be optimized as well. Additionally, more in-depth studies ought to be carried out on the corrosion susceptibility of RTSF and the durability of the AASC containing them.

## Figures and Tables

**Table 1:** Chemical properties of Slag.

Content	SiO_2_	Al_2_O_3_	Fe_2_O_3_	CaO	MgO	SO_3_	K_2_O	Na_2_O	Mn_2_O_3_	TiO_2_	LOI	K_b_	HM
Results	36.50(%)	11.00(%)	0.70(%)	38.50(%)	9.20(%)	0.30(%)	0.60(%)	0.55(%)	1.50(%)	1.50(%)	0.50(%)	1.00(-)	1.61(-)

Note: LOI (Loss on ignition); K_b_ (Basicity coefficient); HM (Hydration modulus).

**Table 2:** Parameters and levels used in the Taguchi experiment design.

Parameters	Designation	Level-1	Level-2	Level-3	Level-4
Binder content (kg/m^3^)	A	350	375	400	425
SH (M)	B	10	12	14	16
Al/Bi ratio	C	0.4	0.45	0.5	0.55
W/S ratio	D	0.35	0.375	0.4	0.425
SS/SH ratio	E	1.75	2	2.25	2.5

**Table 3:** Sixteen sets of mixtures of AASC as per L_16_ orthogonal array and their mix proportions.

Trial Mix	Mix ID	Factors and Values					Concrete Mixture Quantity (kg/m^3^)							
		Binder Content (kg/m^3^)	Molarity of SH (M)	Al/Bi Ratio	W/S Ratio	SS/SH Ratio	Slag	SS	SH	Al	EW	SP	CA	FA
TM1	A1B1C1D1E1	350	10	0.40	0.35	1.75	350	89.1	50.9	140.0	52.1	3.5	943.5	932.5
TM2	A1B2C2D2E2	350	12	0.45	0.38	2.00	350	105.0	52.5	157.5	57.5	3.5	923.5	912.7
TM3	A1B3C3D3E3	350	14	0.50	0.40	2.25	350	121.2	53.8	175.0	63.8	3.5	902.5	891.9
TM4	A1B4C4D4E4	350	16	0.55	0.43	2.50	350	137.5	55.0	192.5	70.7	3.5	880.4	870.1
TM5	A2B1C2D3E4	375	10	0.45	0.40	2.50	375	120.5	48.2	168.8	69.9	3.8	886.8	876.4
TM6	A2B2C1D4E3	375	12	0.40	0.43	2.25	375	103.8	46.2	150.0	92.2	3.8	875.2	865.0
TM7	A2B3C4D1E2	375	14	0.55	0.35	2.00	375	137.5	68.8	206.3	37.3	3.8	898.3	887.8
TM8	A2B4C3D2E1	375	16	0.50	0.38	1.75	375	119.3	68.2	187.5	61.9	3.8	883.9	873.6
TM9	A3B1C3D4E1	400	10	0.50	0.43	2.00	400	133.3	66.7	200.0	75.7	4.0	839.5	829.7
TM10	A3B2C4D3E1	400	12	0.55	0.40	1.75	400	140.0	80.0	220.0	58.7	4.0	844.9	835.0
TM11	A3B3C1D2E4	400	14	0.40	0.38	2.50	400	114.3	45.7	160.0	78.6	4.0	875.2	865.0
TM12	A3B4C2D1E3	400	16	0.45	0.35	2.25	400	124.6	55.4	180.0	61.7	4.0	880.8	870.5
TM13	A4B1C4D2E3	425	10	0.55	0.38	2.25	425	161.8	71.9	233.8	45.6	4.3	838.8	829.0
TM14	A4B2C3D1E4	425	12	0.50	0.35	2.50	425	151.8	60.7	212.5	47.7	4.3	857.5	847.4
TM15	A4B3C2D4E1	425	14	0.45	0.43	1.75	425	121.7	69.5	191.3	99.4	4.3	809.2	799.7
TM16	A4B4C1D3E2	425	16	0.40	0.40	2.00	425	113.3	56.7	170.0	99.9	4.3	829.0	819.3

Note: SS (sodium silicate); SH (sodium hydroxide); Al (alkaline solution); EW (extra water); SP (superplasticizer); CA (coarse aggregates); FA (fine aggregates).

**Table 4:** Experimental results and signal-to-noise (S/N) ratio.

Trial Mix	Mix ID (Combination)	Experimental Results					Signal-to-Noise (S/N) Ratio				
		Slump (mm)	ST (min)		CS (MPa)		Slump	ST		CS	
			Initial	Final	7-day	28-day		Initial	Final	7-day	28-day
TM1	A1B1C1D1E1	45	30	54	35.48	43.59	33.06	29.54	34.65	31.00	32.79
TM2	A1B2C2D2E2	80	34	59	44.10	48.10	38.06	30.63	35.42	32.89	33.64
TM3	A1B3C3D3E3	120	48	83	42.06	47.56	41.58	33.62	38.38	32.48	33.54
TM4	A1B4C4D4E4	150	50	79	42.29	52.49	43.52	33.98	37.95	32.52	34.40
TM5	A2B1C2D3E4	180	33	55	36.78	45.65	45.11	30.37	34.81	31.31	33.19
TM6	A2B2C1D4E3	190	35	63	36.48	44.96	45.58	30.88	35.99	31.24	33.06
TM7	A2B3C4D1E2	55	52	90	47.41	59.44	34.81	34.32	39.08	33.52	35.48
TM8	A2B4C3D2E1	90	50	85	46.26	54.64	39.08	33.98	38.59	33.30	34.75
TM9	A3B1C3D4E1	240	49	84	36.63	50.62	47.60	33.80	38.49	31.28	34.09
TM10	A3B2C4D3E1	200	54	88	44.89	58.00	46.02	34.65	38.89	33.04	35.27
TM11	A3B3C1D2E4	130	34	60	41.64	55.82	42.28	30.63	35.56	32.39	34.94
TM12	A3B4C2D1E3	65	37	63	46.19	61.53	36.26	31.36	35.99	33.29	35.78
TM13	A4B1C4D2E3	225	41	72	38.63	52.38	47.04	32.26	37.15	31.74	34.38
TM14	A4B2C3D1E4	115	38	64	44.78	56.81	41.21	31.60	36.12	33.02	35.09
TM15	A4B3C2D4E1	260	40	68	41.29	53.54	48.30	32.04	36.65	32.32	34.57
TM16	A4B4C1D3E2	210	33	57	41.37	54.20	46.44	30.37	35.12	32.33	34.68

Note: ST (setting time); CS (compressive strength).

**Table 5:** Grey relational analysis results.

Mix No	Normalized S/N Ratio					Deviation Sequences					Grey Relational Coefficient (GRC)					GRG	
	Slump	ST		CS		Slump	ST		CS		Slump	ST		CS		Value	Rank
		Initial	Final	7-Day	28-Day		Initial	Final	7-Day	28-Day		Initial	Final	7-Day	28-Day		
TM1	0.00	0.00	0.00	0.00	0.00	1.00	1.00	1.00	1.00	1.00	0.33	0.33	0.33	0.33	0.33	0.33	16
TM2	0.33	0.21	0.17	0.75	0.29	0.67	0.79	0.83	0.25	0.71	0.43	0.39	0.38	0.67	0.41	0.45	13
TM3	0.56	0.80	0.84	0.59	0.25	0.44	0.20	0.16	0.41	0.75	0.53	0.71	0.76	0.55	0.40	0.59	8
TM4	0.69	0.87	0.74	0.61	0.54	0.31	0.13	0.26	0.39	0.46	0.61	0.79	0.66	0.56	0.52	0.63	5
TM5	0.79	0.16	0.04	0.12	0.13	0.21	0.84	0.96	0.88	0.87	0.70	0.37	0.34	0.36	0.37	0.43	15
TM6	0.82	0.26	0.30	0.10	0.09	0.18	0.74	0.70	0.90	0.91	0.74	0.40	0.42	0.36	0.35	0.45	14
TM7	0.11	0.94	1.00	1.00	0.90	0.89	0.06	0.00	0.00	0.10	0.36	0.89	1.00	1.00	0.83	0.82	2
TM8	0.40	0.87	0.89	0.92	0.66	0.60	0.13	0.11	0.08	0.34	0.45	0.79	0.82	0.86	0.59	0.70	3
TM9	0.95	0.83	0.86	0.11	0.43	0.05	0.17	0.14	0.89	0.57	0.92	0.75	0.79	0.36	0.47	0.66	4
TM10	0.85	1.00	0.96	0.81	0.83	0.15	0.00	0.04	0.19	0.17	0.77	1.00	0.92	0.73	0.74	0.83	1
TM11	0.60	0.21	0.21	0.55	0.72	0.40	0.79	0.79	0.45	0.28	0.56	0.39	0.39	0.53	0.64	0.50	12
TM12	0.21	0.36	0.30	0.91	1.00	0.79	0.64	0.70	0.09	0.00	0.39	0.44	0.42	0.85	1.00	0.62	6
TM13	0.92	0.53	0.56	0.29	0.53	0.08	0.47	0.44	0.71	0.47	0.86	0.52	0.53	0.41	0.52	0.57	9
TM14	0.53	0.40	0.33	0.80	0.77	0.47	0.60	0.67	0.20	0.23	0.52	0.46	0.43	0.72	0.68	0.56	10
TM15	1.00	0.49	0.45	0.52	0.60	0.00	0.51	0.55	0.48	0.40	1.00	0.49	0.48	0.51	0.55	0.61	7
TM16	0.88	0.16	0.11	0.53	0.63	0.12	0.84	0.89	0.47	0.37	0.80	0.37	0.36	0.52	0.58	0.53	11
Mean GRC											0.62	0.57	0.56	0.58	0.56		

Note: ST (setting time); CS (compressive strength); GRG (Grey relational grade).

**Table 6:** Main effect table for the grey relational grade.

Parameters	Mean Grey Relational Grade				Effect	
	Level-1	Level-2	Level-3	Level-4	Delta	Rank
A: Binder content	0.50	0.60	**0.65**	0.57	0.15	2
B: SH	0.50	0.58	**0.63**	0.62	0.13	3
C: Al/Bi ratio	0.45	0.53	0.63	**0.71**	0.26	1
D: W/S ratio	0.58	0.56	**0.60**	0.59	0.04	5
E: SS/SH ratio	**0.62**	0.61	0.56	0.53	0.09	4

**Table 7:** ANOVA results of grey relational grade.

Factor	DOF	SOS	MS	Contribution (%)
A: Binder content	3	0.0474	0.01581	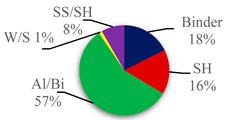
B: SH	3	0.0431	0.01436	
C: Al/Bi	3	0.1535	0.05118	
D: W/S	3	0.0034	0.00112	
E: SS/SH	3	0.0224	0.00745	
Error	-	-	-	
Total	15	0.2698		

Note: DOF (degree of freedom); SOS (sum of square); MS (mean square).

**Table 8:** Results of confirmation experiment.

AASC Outputs	Initial Condition	Optimum Parameters Condition	
		Prediction	Experiment
	A3B2C4D3E1	A3B3C4D3E1	A3B3C4D3E1
Slump (mm)	200		195
Initial ST (min)	54		56
Final ST (min)	88		95
7-day CS (N/mm^2^)	44.89		45.67
28-day CS (N/mm^2^)	58		59.4
Grey relational grade	0.83	0.885	0.876

Note: ST (setting time); CS (compressive strength).

**Table 9:** Mix proportion of fiber-reinforced alkali-activated slag-based concrete (FR-AASC) and corresponding slump values.

Mix No.	Mixture ID	RTSF Volume Fraction (%)	Binder Content (kg/m^3^)	Molarity of SH (M)	Al/Bi Ratio	W/S Ratio	SS/SH Ratio	Slump (mm)
TM17	S0	0	400	14	0.55	0.4	1.75	195
TM18	S0.2	0.2	400	14	0.55	0.4	1.75	180
TM19	S0.4	0.4	400	14	0.55	0.4	1.75	140
TM20	S0.6	0.6	400	14	0.55	0.4	1.75	90

Note: RTSF (recycled tire steel fiber).

## Data Availability

The data that support the finding of this study are available from the corresponding author upon request.
